# The goitre rate, its association with reproductive failure, and the knowledge of iodine deficiency disorders (IDD) among women in Ethiopia: Cross-section community based study

**DOI:** 10.1186/1471-2458-7-316

**Published:** 2007-11-08

**Authors:** Cherinet Abuye, Yemane Berhane

**Affiliations:** 1 Food and Nutrition Research Department, Ethiopian Health and Nutrition Research Institute, Addis Ababa, Ethiopia; 2 Department of Community Health, Faculty of Medicine, Addis Ababa University, Addis Ababa, Ethiopia

## Abstract

**Background:**

Iodine deficiency is severe public health problem in Ethiopia. Although urinary iodine excretion level (UIE) is a better indicator for IDD the goitre rate is commonly used to mark the public health significance. The range of ill effect of IDD is however beyond goitre in Ethiopia. In this study the prevalence of goitre and its association with reproductive failure, and the knowledge of women on Iodine Deficiency were investigated.

**Methods:**

A cross-section community based study was conducted during February to May 2005 in 10998 women in child bearing age of 15 to 49 years. To assess the state of iodine deficiency in Ethiopia, a multistage "Proportional to Population Size" (PPS) sampling methods was used, and WHO/UNICEF/ICCIDD recommended method for goitre classification.

**Results:**

Total goitre prevalence (weighted) was 35.8% (95% CI 34.5–37.1), 24.3% palpable and 11.5% visible goitre. This demonstrates that more than 6 million women were affected by goitre.

Goitre prevalence in four regional states namely Southern Nation Nationalities and People (SNNP), Oromia, Bebshandul-Gumuz and Tigray was greater than 30%, an indication of severe iodine deficiency. In the rest of the regions except Gambella, the IDD situation was mild to moderate. According to WHO/UNICEF/ICCIDD this is a lucid indication that IDD is a major public health problem in Ethiopia. Women with goitre experience more pregnancy failure (X^2 ^= 16.5, p < 0.001; OR = 1.26, 1.12 < OR < 1.41) than non goitrous women. Similarly reproductive failure in high goitre endemic areas was significantly higher (X^2 ^= 67.52; p < 0.001) than in low. More than 90% of child bearing age women didn't know the cause of iodine deficiency and the importance of iodated salt.

**Conclusion:**

Ethiopia is at risk of iodine deficiency disorders. The findings presented in this report emphasis on a sustainable iodine intervention program targeted at population particularly reproductive age women. Nutrition education along with Universal Salt Iodization program and iodized oil capsule distribution in some peripheries where iodine deficiency is severe is urgently required.

## Background

According to the WHO, Iodine Deficiency Disorders are among the major public health problems of the world, particularly of pregnant and young women in many developing countries [1]. Low level of thyroid hormones in the body due to lack of adequate iodine in foods and drinks is responsible for iodine deficiency disorders. Iodine deficiency causes a wide range of health related problems that are collectively called Iodine Deficiency Disorders (IDD) [2,3]. When goitre prevalence is severe or exceeds 30% in a given area, cretinism may affect up to 5 to 15% of the population [1]. It has been known that as severity of iodine deficiency increase the occurrence of poor pregnancy out come such as miscarriage, stillbirth, and increased infant mortality is more likely [4-7]. Several experimental studies revealed that, reproductive failure could be corrected by iodine supplementation in goitre endemic areas [5].

In Ethiopia IDD has been recognized as a public health problem for many decades [8-13]. A recent study [14] that did both clinical and biochemical assessment has confirmed that the situation of IDD has deteriorated. In high goitre endemic regions the prevalence of goitre in children was greater than 30% while in less endemic regions it was less than 15%. The reported median national UIE concentration of 24.5 μg/L is far below the WHO/UNICEF/ICCIDD cut-off point 100 μg/L or above to indicate adequate iodine intake in the population. More than 83% of the study population had UIE less than 100 μg/L, of these 45% had UIE level less than 20 μg/L revealing severe iodine deficiency while 22.6% had moderate iodine deficiency, UIE between 20.1 and 50 μg/L.

Reported studies from Ethiopia have been mainly on the goitre prevalence. Other effects of iodine deficiency particularly the negative effects on reproductive outcome were not studied. Besides establishing baseline national information on goitre rate and knowledge of iodine deficiency among women, this study aimed to assess its association with pregnancy outcome in Ethiopia.

## Methods

A cross-sectional community based study was conducted at the national level in Ethiopia during February to May 2005, among women age 15 – 49 years. Women of this age group are recommended for the assessment of the severity of iodine deficiency and the link between iodine deficiency and poor pregnancy outcome because of their dramatic response to the inadequate intake of iodine.

Ethiopia, situated in the horn of East Africa, is mountainous where the top layer of the soil in many parts has been eroded for decades leading to leaching away of nutrient including iodine [15].

According to the Federal Ministry of Health of Ethiopia 2004 [16], the country's estimated population was more than 73 million. Majority, more than 85% are rural dwellers depending on subsistent agricultural economy. The staple crops are teff (Eragrostis tef) and cereals in the North and Central part, enset (Ensete ventricosum), cassava (Manihot esculenta), maize (Zea mays), cereals and root crops in the South and Southwest, and sorghum and maize in the East of the country.

Out of 11 regional states, 10 were included in the study. In one of the regions only regional capital and surrounding areas were assessed because other parts were inaccessible for security reason. In the selected cluster (a cluster is equivalent to the smallest administrative unit as defined by the Government and is commonly known as "kebele"), only women in child bearing age, married and volunteer to participate in the interview were included.

Multistage cluster sampling methods was applied to select study population. A total of 10998 women, thirty per cluster, were included in the study. Thirty clusters per regional state were selected by assigning probability proportional to the population size. In each regional state, cumulative populations were calculated and attributed number assigned. The sampling interval was then calculated by dividing the total number of study population with the number of clusters (thirty). A random number was drawn using a random number table. The first cluster selected based on this number. To select the others clusters the sampling interval was added sequentially to the random number till all thirty clusters were selected.

In four large and densely populated regional states namely Amhara, Oromia, Southern Nations Nationalities and peoples (SNNP) and Tigray, where more than 80% of the country's population live the study was conducted both in high and low land villages of the selected areas. The sample size taken in each of these regions was nearly twice that of the sample size in the rest of the states. The initial aim was to assess IDD in the two eco-zones independently. However, because of lack of separately delineated lowland and highland population; results were not compared by topography. Besides, in some of the regions due to non-respondents and security reason expected sample size was not obtained.

In each cluster households were selected by locating the centre of the locality/kebele and spinning a pen and proceeding to the direction that the pen pointed to. Household along the direction to the boundary of the locality was counted and numbered. A random number selected which determined the starting point, the first house selected, to examine and interview eligible women. Then the subsequent households in the identified direction were visited until thirty women per cluster were obtained for thyroid size estimation and interview. Those women who were not present at the time of the survey were revisited, interviewed and examined clinically for goitre.

Doctors and nurses were recruited and trained for one week to serve as data collectors. The training was focused on how to do standardized clinical examination of goitre and on interview techniques. Doctors were assigned to examine thyroid size of a group of subjects selected from goitre endemic village. The data from each examiner was compiled and the variation was assessed. The standardization procedure continued until inter-observer variation was negligible. In the actual study, team leaders did random counter checking on clinical examination everyday. Doctors undertook the clinical examination, and nurse the interviews.

Reproductive failure is defined as a history of having at least one or more miscarriage and or stillbirth. Retrospective information on reproductive failure and knowledge on IDD such as causes of goitre, health problems associated with goitre, importance of iodated salt, iodated salt handling and other health and demographic information were collected using structured household questionnaire. The nurses administered the questionnaire to women. Data were collected by house-to-house visit.

Supervisors who are responsible to coordinate and lead the overall activities reviewed the questionnaires on the spot to ensure completion and accuracy. Every evening survey group had meeting to discuss the experience of the day and plan for the next day.

Physical examination of the thyroid gland was done to assess goitre rate using the WHO/UNICEF/ICCIDD classification scheme [17] as follows:

Grade 0: None or no goitre (palpable or visible)

Grade 1 or palpable:  A goitre that is palpable but not visible when the neck is in the normal position, (i.e. the thyroid is not visibly enlarged).

Grade 2 or visible:  A swelling in the neck that is clearly visible when the neck is in a normal position and is consistent with an enlarged thyroid when the neck is palpated.

Total Goitre Rate (TGR): Sum of goitre grades 1 and 2.

Data was entered into a computer and analyzed using SPSS for windows, version 10, 1997. X^2 ^– test was used to assess level difference in reproductive failure between high (seven) and less endemic (three) regions. The grouping of endemic regions was done based on the median urinary iodine level determined by another component of the study (reference 14). Accordingly 7 of the 10 regions studied were grouped to high and the remaining 3 to low endemic regions

Similarly history of reproductive failure was compared between goitrous and non goitrous women. Goitre prevalence of each regional state was weighted according to total population size of the region. Then the overall (national weighted rate of goitre with 95% confidence interval (CI) was given to make regional distribution of goitre nationally representative.

### Informed consent

In each regional state, regional administration approved this study. All women gave their informed consent prior to their inclusion in interview and clinical examination.

## Results

The mean age of the 10988 women assessed for goitre was 31.8 ± 6.9 years. About 80% of the women in the study were from rural areas.

Table 1 shows palpable, visible and total goitre rates (TGR) with 95% confidence interval by regional states. TGR in four of the regions namely SNNP, Benshangul-Gumuz, Tigray and Oromiya was greater than 30%, with the maximum of about 60% in SNNPR, an indication of severe iodine deficiency in the states. In the rest of the regional states except Gambella where the rate is 1.4% the goitre prevalence ranged from 5 to 29.9%. According to the WHO/UNICEF/ICCIDD classifications, this is mild to moderate degree of iodine deficiency.

**Table 1:** Goitre rate of women by regional states

Region	Number examined.	Palpable % (95% CI)	Visible % (95% CI)	TGR% (95% CI)
Amhara	1637	20.2% (17.4,23.0)	8.6% (6.7,10.5)	28.8% (25.7,31.9)
Oromia	1816	20.0% (17.4,22.6)	11.3% (9.2,13.4)	31.3% (28.3,34.3)
Tigray	1796	22.2% (19.5,24.9)	13.4% (11.2,15.5)	35.6% 32.5,38.7)
SNNPR	1702	43.2% (39.9,46.5)	17.7% (15.1,20.3)	59.9% (57.6,64.2)
Addis Ababa	804	15.0% (11.5,18.5)	7.3% (4.8,9.8)	22.3% (18.2,26.4)
Afar	728	14.1% (10.5,17.7)	1.5% (0.2,2.8)	15.6% (11.9,19.3)
Benshangul Gumuz	888	21.8% (18.0,25.6)	15.5% (11.8,19.2)	37.3% (32.8,41.8)
Dire Dawa	749	11.5% (8.3,14.7)	0.9% (0,1.8)	12.4% (9.1,15.7)
Harari	730	4.1% (2.1,6.1)	2.6% (1.0,4.2)	6.7% (4.0,9.3)
Gambella*	148	1.4% (0,4.1)	-	1.4% (0,4.1)
Total goitre rate (Weighted)	10998	24.3% (23.2,25.4)	11.5% (10.7,12.3)	35.8% (34.5,37.1)

*Under represented because of security reason

Weighted total goitre rate in 15 to 49 year-old women was 35.8% (95%, CI = 34.5 – 37.1), 24.3% palpable and 11.5% visible goitre (table 1). This indicates that about 6 million women in this age category were affected by goitre in Ethiopia.

Table 2 presents goitre rate among women in different age categories, residence and physiological status (non-pregnant non-lactating and pregnant and or lactating). Goitre rate decreased as the age categories increased. The highest (37%) total goitre rate was found in the 15–24 years old group. Rural women were significantly (P < 0.001) affected by goitre than women in urban or semi-urban settings. Concerning physiological status of the women, of the 4796 pregnant and or lactating women, 36% had total goitre prevalence while in 6122 non-pregnant and non-lactating the rate was 28%. Goitre was significantly (X^2 ^= 68.8; p < 0.001) frequent in pregnant and or lactating women than non-pregnant and non-lactating counter parts.

**Table 2:** Goitre rate of women by age categories, residence and physiological status

Characteristics	Categories	No. of women	Goitre rate n (%)
Age	15–24	973	360(37)
	25–34	5934	1920(32.5)
	35–49	3950	1150(29.1)
Residence	Semi-urban or urban	2431	323 (13.3) *
	Rural	8567	3134 (36.6)
Pregnant/lactating	Yes	4796	1720(36)
	No	6122	1740(28)**

* X^2 ^= 476.8, p < 0.0001

* * X^2 ^= 68.8, p < 0.0001

The association of reproductive failure with goitre and goitre endemicity was examined and the results are given in table 3. Two groups of women, (goitrous and non-goitrous) examined for goitre grades were compared for history of pregnancy failure.

**Table 3:** Number of women with reproductive failure (stillbirth &/or miscarriage) according to goitre rate and goitre endemic sites

	Stillbirth and/or miscarriage		
Characteristics	n (%)	X^2^, (P-value)	OR, (95% CI)

Women with goitre (n = 3487)	584 (17%)	16.5 (<0.001)	1.26 (1.12,1.41)
Women without goitre (n = 7515)	1037 (14%)		
High goitre endemic regions (n = 9489)	1481 (16%)	67.2 (<0.001)	2.20 (1.81,2.68)
Less goitre endemic regions (n = 1585)	123 (7.8%)		

Of the 3487 women with goitre 16.7% had history of one or more reproductive failure in the form of miscarriage and or stillbirth while among 7515 women without goitre 13.8% reported to have reproductive failure. The presence of goitre was statistically associated (X^2 ^= 16.5, p < 0.001; OR = 1.26, 1.12 < OR < 1.41) with reproductive failure.

An attempt was also made to determine whether pregnancy outcome was associated with endemicity of goitre (table 3). Regional states were grouped according to their endemicity of goitre. High endemic regions had goitre rate of 35.4% while less endemic had 11.2%. This shows that women in high goitre endemic regions had greater (X^2 ^= 67.52; p < 0.001) risk of reproductive failure than those in less goitre endemic regions in the country.

Further analysis was done to assess the relationship between pregnancy failure (stillbirth) and presence goitre. Of 6914 women without goitre, 435 (6.3%) had stillbirth where as the rate was 8.5% (271/3174) in women with goitre indicating that stillbirth is significantly linked to the presence of goitre in women (X^2 ^= 16.9, p < 0.001; OR = 1.39, 1.18 < OR < 1.63)

Knowledge on IDD and use of iodated salt was assessed in the survey households. In all the regional states except Addis Ababa, more than 90% of the study women did not know the importance of iodated salt (table 4). Similarly apart few in Tigray and Addis Ababa, majority (> 90%) had no understanding about the causes of iodine deficiency.

**Table 4:** Knowledge on Iodine Deficiency Disorders

Region	Total	Do you know the importance of iodated salt		Do you know the cause for goitre	
		Yes %	No %	Yes %	No %

Amhara	1620	3.5	96.3	3.7	96.3
Oromia	1717	2.4	97.6	6.7	93.3
Tigray	1769	9.3	90.7	19.5	80.5
SNNPR	1677	1.5	98.5	4.3	95.7
Addis Ababa	830	31.6	68.4	27.2	72.8
Afar	730	-	100	2.6	97.4
Benshagul Gumuz	882	6.8	93.2	7.2	92.8
Dire Dawa	751	4.4	95.6	4.0	96.0
Harari	776	8.7	91.3	11.7	88.3
Gambella*	142	2.1	97.9	4.2	95.8
Average			93%		91%

## Discussion

The findings of iodine deficiency rate of 35.8% signify that Ethiopia is severely affected by iodine deficiency. Severe iodine deficiency is significantly associated with reproductive failure. In this study, women with goitre and women living in highly goitre endemic areas were more likely to experience reproductive failure in the form of miscarriage and stillbirth than those living in less endemic regions or women without goitre. Goitre was more frequent in young women and in rural areas. The association between goitre and reproductive failure found in the current survey is agreement with what has been reported previously [5,6].

The regional states that were severely affected (>30% TGR in women), were the Southern Nations, Oromia, Tigray and Beshangul-Gumuz regional states. About 60% of the country's population lives in these regional states. TGR in the rest of the regions except Gambella was between 5 – 29.9%. According to WHO/UNICEF/ICCIDD [17] a total goitre rate of 5% is an indicative of a public health risk of adverse functional consequences and when higher than 30% the problem is severe.

Even though the problem of iodine deficiency is serious, no control program has been implemented in Ethiopia except the 1988 salt iodization program initiated at the red sea port of Asab, which was discontinued after few years of operation due to political instability in the area. Currently because of adequate production of salt within the country, the Federal Ministry of Health (FMOH) with other stake holders has pledge to implement the Universal Salt Iodization (USI) program in Ethiopia.

The draft legislation for Universal Salt Iodization (USI) Program, with implementing regulations is under review. The legislation bans importation, production and distribution of non-iodated salt in the country.

In line with the present survey, several studies [8-13] including a recent national study [14] undertaken to assess iodine deficiency in children age 6 to 12 years using both clinical and biochemical data have revealed that iodine deficiency is a serious public health problem in Ethiopia. The trends as well as goitre rate in the current and previously reported surveys were more or less similar. Pregnancy-associated severe iodine deficiency limits ones intellectual capacity, occupational choices, economic development and future earning [18-20]. Regarding cause for goitre, previous studies [21] revealed that besides iodine deficiency goitrogenic factors from cassava consumption contribute to the high prevalence of goitre and iodine deficiency associated health problems in Ethiopia.

Cretinism rate increases in a given area when goitre rate is above 30 percent [3,18]. Mentally and physically retarded subjects shown in a documentary film [IDD in Ethiopia 2005] produced by former Ethiopian Nutrition Institute and UNICEF depicts severity and wide range of IDD in Ethiopia. Besides goitre and cretinism iodine deficiency is a well-known cause for several disorders such as poor pregnancy outcome because women in reproductive age are more vulnerable to iodine deficiency.

Several experimental studies done in animal species have shown that iodine deficiency has dramatic effect on reproductive failure and goitre. In sheep during pregnancy, iodine deficiency increased the number of abortions and stillbirth [7]. Twenty-one percent of the iodine deficient ewes had reproductive failure, compared with 4% of the control. Studies in hypothyroid guinea pig (produced by surgical removal of the thyroid) revealed a three to four-fold increase in abortion and stillbirth, which practically could be eliminated by replacement therapy with thyroxin during pregnancy [7].

Iodine deficiency causes spontaneous abortion, stillbirth, and neonatal deaths in women living in different iodine deficient parts of the world [5-7]. Iodine supplementation to pregnant women in New Guinea reduced poor pregnancy outcome from 24% to 16% [5].

We also assessed IDD knowledge of women while investigating iodine deficiency related problems because this should be one of the components to be addressed for the success of the upcoming intervention program in the country. As most women did not have knowledge on IDD, the link between pregnancy outcome and knowledge of IDD was not assessed. Over 90% of the women in all regional states did not know the importance of iodized salt and cause of goitre except in Addis Ababa and Tigray regions where relatively better proportion of women had knowledge about IDD. In an earlier series it was also observed that in many parts of Ethiopia goitre is known only when it is visible and the community seeks medication usually when goitre becomes symptomatic.

## Conclusion

Iodine deficiency has several dimensions to be addressed in Ethiopia. Goitre prevalence in the Southern Nations Nationalities and Peoples, Oromia, Benishangul-Gumuz, and Tigray regional states indicates the existence of severe iodine deficiency. According to WHO/UNICEF/ICCIDD this is a clear indication that iodine deficiency is a major public health problem in Ethiopia and the country is at an increasing risk of IDD. Women with goitre experience more pregnancy failure than women without goitre and the reproductive failure is significantly higher in high goitre endemic areas. The evidence of the current survey shows that the problem is rampant still severe and is significantly associated with poor pregnancy outcome. Most women do not know IDD, the cause of iodine deficiency and the importance of iodized salt. The situation is apparently alarming. The findings presented in this report therefore emphasize on a population particularly reproductive-age women targeted sustainable iodine intervention program. Integrated approaches including a sustainable USI program, iodized oil capsule distribution in some peripheries of the country and nutrition education to increase community awareness are highly recommended.

## Competing interests

The author(s) declare that they have no competing interests.

## Authors' contributions

CH carried out Project design, analysis, interpretation and preparation of manuscript. YB involved in project design/supervision, interpretation of results and revision of the manuscript.

## Pre-publication history

The pre-publication history for this paper can be accessed here:


